# Supplementary material for: Metabolomic Analysis of Respiratory Epithelial Lining Fluid in Patients with Chronic Obstructive Pulmonary Disease—A Systematic Review
Source: Cells. 2023 Mar 8;12(6):833. doi: 10.3390/cells12060833 (PMC10047085; doi:10.3390/cells12060833)
Supplement: Supplementary file 1 [file cells-12-00833-s001.zip › cells-2160145-supplementary.pdf]

Supplementary material

Table S1. Phenotypes of COPD

**Phenotype**	**Diagnosis [1]**	**Prognosis [2,3]**	**Phenotype-specific management [3,4]**
Predominant emphysema	Presence of emphysema confirmed on HRCT<br><br>Key molecules [5]<br>→ Leucine<br>→ Lysine	Rapid decline in lung function<br><br>Higher mortality risk	Standard symptomatic treatment of COPD<br><br>Causal treatment does not exist
Chronic bronchitis	Productive cough more than 3 months per year in two or more consecutive years	Slower decline in lung function<br><br>Lower mortality risk	Phosphodiesterase-4 inhibitors
Frequent exacerbations	2 or more exacerbations per year<br><br>Key molecules [6]<br>→ Undecane<br>→ Tetramethyloctane<br>→ Methanoazulene<br>→ Naphthalene	Rapid decline in lung function<br><br>Higher mortality risk<br><br>Predictive biomarkers of exacerbation [7]<br>→ Sialic acid<br>→ Hypoxanthine	Inhaled glicocorticosteroids<br><br>Vaccinations
Eosinophilic phenotype	Sputum eosinophilia >3%<br><br>Key molecules [6]<br>→α-methylstyrene<br>→ Cyclohexenol<br>→ Benzofuran<br>→ Decane<br>→ Biphenyl	Rapid decline in lung function<br><br>Higher mortality risk	Inhaled glicocorticosteroids

1. Manian, P. Chronic obstructive pulmonary disease classification, phenotypes and risk assessment. *Journal of thoracic disease* **2019**, *11*, S1761.
2. Brat, K.; Svoboda, M.; Hejduk, K.; Plutinsky, M.; Zatloukal, J.; Volakova, E.; Popelkova, P.; Novotna, B.; Engova, D.; Franssen, F.M. Introducing a new prognostic instrument for long-term mortality prediction in COPD patients: the CADOT index. *Biomedical Papers of the Medical Faculty of Palacky University in Olomouc* **2021**, *165*.
3. Adeloye, D.; Song, P.; Zhu, Y.; Campbell, H.; Sheikh, A.; Rudan, I. Global, regional, and national prevalence of, and risk factors for, chronic obstructive pulmonary disease (COPD) in 2019: a systematic review and modelling analysis. *The Lancet Respiratory Medicine* **2022**, *10*, 447-458.
4. Venkatesan, P. GOLD COPD report: 2023 update. *The Lancet Respiratory Medicine* **2023**, *11*, 18.
5. Halper-Stromberg, E.; Gillenwater, L.; Cruickshank-Quinn, C.; O'Neal, W.K.; Reisdorph, N.; Petrache, I.; Zhuang, Y.H.; Labaki, W.W.; Curtis, J.L.; Wells, J., et al. Bronchoalveolar Lavage Fluid from COPD Patients Reveals More Compounds Associated with Disease than Matched Plasma. *Metabolites* **2019**, *9*, doi:10.3390/metabo9080157.
6. Basanta, M.; Ibrahim, B.; Dockry, R.; Douce, D.; Morris, M.; Singh, D.; Woodcock, A.; Fowler, S.J. Exhaled volatile organic compounds for phenotyping chronic obstructive pulmonary disease: a cross-sectional study. *Respiratory research* **2012**, *13*, doi:10.1186/1465-9921-13-72.
7. Esther Jr, C.R.; O’Neal, W.K.; Anderson, W.H.; Kesimer, M.; Ceppe, A.; Doerschuk, C.M.; Alexis, N.E.; Hastie, A.T.; Barr, R.G.; Bowler, R.P. Identification of sputum biomarkers predictive of pulmonary exacerbations in COPD. *Chest* **2022**, *161*, 1239-1249.
